# What's the norm in normalization? A frightening note on the use of RT-qPCR in the livestock science

**DOI:** 10.1016/j.gene.2018.100003

**Published:** 2018-12-23

**Authors:** Sebastiano Busato, Matteo Mezzetti, Paul Logan, Nicolas Aguilera, Massimo Bionaz

**Affiliations:** a Department of Animal and Rangeland Sciences, Oregon State University, Corvallis, OR 97331, United States of America; b Istituto di Zootecnica, Facoltà di Agraria, Università Cattolica del Sacro Cuore, 29122 Piacenza, Italy

**Keywords:** ACTB, Beta-actin, cDNA, Complementary DNA, EIF3K, Eukaryotic Translation Initiation Factor 3 Subunit K, GAPDH, Glyceraldehyde 3-phosphate dehydrogenase, ICG, Internal Control Gene(s), IF, Journal Impact Factor, MIQE, Minimum Information of the publication of RT-qPCR data, NAL, Article that did not use an algorithm to validate the results, PPIA, Peptidylprolyl Isomerase A, RIN, RNA Integrity Number, RNA18S, 18S ribosomal RNA, RPL19, Ribosomal Protein L19, RPS9, Ribosomal Protein S9, RT-qPCR, Reverse-Transcription quantitative Polymerase Chain Reaction, TBP, TATA-Box Binding Protein, UXT, Ubiquitously Expressed Gene, YAL, Article using an algorithm to validate the results, RT-qPCR, Gene expression, Normalization, Livestock

## Abstract

Reverse-Transcription quantitative PCR (**RT-qPCR**) provides a valuable tool to study gene expression with exquisite sensitivity. To retain its inferential power, user-introduced technical variability must be reduced and accounted for. Selecting a set of stably expressed internal control genes (**ICG**), validated for each experimental condition/sample set, is widely accepted as a reliable way to normalize RT-qPCR data and account for said variability. Despite significant efforts in establishing standardized and resource-efficient normalization approaches, numerous recent reports have underlined deficiencies in the state of RT-qPCR normalization. Livestock science has benefitted tremendously from the use of RT-qPCR; however, the issue of lack of proper normalization likely affects this discipline as well. We thus decided to determine whether this is true, and to which extent. We conducted an in-depth analysis of all (225) RT-qPCR articles published in the six most prominent livestock journals in the field from 2013 to 2017. A quantitative scale was constructed, and values were assigned to each article based on the number of ICG used, the use of a publicly available algorithm to assess the reliability of ICG, and the reporting of pertinent information related to ICG (ranges from 0 = total noncompliance - to 100 = total compliance). Out of the surveyed group, only 10.7% of the publications obtained a score of 100, while the largest group (*n* = 158) was represented by articles that scored 0. Subdividing articles based on whether an algorithm to validate ICG was used (**YAL**) or not (**NAL**) revealed the use of a larger number of ICG to normalize RT-qPCR in the YAL group compared to NAL (1.4-fold more, 95% C.I.: 1.11–1.84) and was closer to the “gold standard” of three ICG. Using an algorithm also increased the diversity of ICG and significantly reduced the use of *RNA18S*, whose suitability as ICG has been thoroughly debated. These remarkably low normalization standards are likely to generate questionable results that can severely hinder the advance of transcriptomic studies in livestock science and related fields.

## Highlights

• Subpar normalization of reverse transcription qPCR was reported for the five major livestock journals over the period 2013-2017
• The use of algorithms for validation led to a greater number of ICG used, and greater variety of ICGs
• The issue could be solved by editors by enforcing the use of the standardized MIQE guidelines for RT-qPCR

## 1 Introduction

The widespread adoption of gene expression analyses has broadened our insights on the transcriptome and facilitated its organization in easily accessible databases, expediting the spread of follow-up and consecutive studies. While the development of massively parallel sequencing techniques has allowed for cost-effective whole-transcriptome studies (Wang et al., 2009), Reverse-Transcription Quantitative PCR (**RT-qPCR**) still offers unrivaled sensitivity in small to medium gene pool sizes.

Akin in its fundamental principles to legacy PCR, RT-qPCR introduces a preliminary step in which isolated RNA is reverse-transcribed (**RT**) to complementary DNA (**cDNA**); the latter is then amplified by a polymerase and quantified in real-time via a fluorescent probe or dye. Despite its clear advantage over gel-based detection methods, the quantitative nature of this assay renders it considerably susceptible to exogenous sources of variation, both intra- and inter-assay. When left unaddressed, differences in initial sample size, RNA recovery, varying rRNA/mRNA ratios, and RT and amplification efficiency, can and often will taint the results, insinuating relative expression changes that are not representative of a true biological response (Bustin, 2010).

One could argue that, in order to minimize and account for this extrinsic source of variation, every research article where RT-qPCR is employed should have an extensive ‘Materials and Methods’ section, carefully detailing all the conditions that might affect the reliability and reproducibility of the assay. In an attempt to introduce standardize practices and comprehensive reporting Bustin et al. in 2009 established the Minimum Information for publication of Quantitative real-time PCR Experiments (**MIQE** guidelines (Bustin et al., 2009)), followed by a lighter and more practical version (MIQE précis (Bustin et al., 2010)) the following year. *Ignorantia juris non excusat*, nor would the allegation that time and resources are unfairly wasted to comply with the MIQE guidelines, since most of the information deemed “Essential” by MIQE are criteria that the researchers should already abide by (e.g. sample selection, assay optimization conditions and the like (Bustin, 2010; Taylor et al., 2010)). However, a 2013 review of >1700 publications revealed a lack of comprehensive reporting in the vast majority of the cases, and very low compliance with the MIQE guidelines (Bustin et al., 2013). For all those studies that do not report an in-depth description of the reagents and assay conditions, it is close to impossible to determine whether the observed changes in gene expression are caused by an actual biological effect or by externally-introduced technical variation, often resulting in misleading results and unsubstantiated conclusions (Udvardi et al., 2008; Ramakers et al., 2003; Bustin, 2000).

Of all the criteria listed on the guidelines, the issue of data normalization deserves special attention. RT-qPCR-based endeavors should account for exogenous sources of variation by referring to a series of stable reference genes (or internal control genes - **ICG**), and “scaling” expression data to these. MIQE clearly expresses that normalizing using a single reference gene is unacceptable “unless the investigators present clear evidence for the reviewers that confirms its invariant expression” and that “the optimal number and choice of reference genes must be experimentally determined” (Bustin et al., 2009). As a matter of fact, many proof-of-concept experiments have pointed out throughout the years (Kadegowda et al., 2009a; Martino et al., 2011; Everaert et al., 2011) that expression levels and stability of a specific gene are extremely variable among samples, tissues and species; the wrong choice of ICG thus completely distorts the results, often generating conflicting conclusions from similar studies, undermining a priori any effort at reproducibility.

Conversely, reliable and consistent RT-qPCR normalization is often based on one of two algorithms: the first implies the validation of a pool of non-coregulated ICG, where pairwise variation is used as a gauge of technical variability (Vandesompele et al., 2002); the second uses an ANOVA-like approach, where stability of a gene is determined throughout all sample groups and subgroups (Andersen et al., 2004). Despite technical differences between the two, both techniques are compliant with MIQE, as they imply experimental validation of ICG (De Spiegelaere et al., 2015; Nolan et al., 2006). Software packages to aid in this purpose are easily accessible such as geNorm (Vandesompele et al., 2002), NormFinder (Andersen et al., 2004) and BestKeeper (Pfaffl et al., 2004) and their respective publications have been cited plentifully (7753, 2991, and 2028 citations in Web of Science, respectively, as of 24th August 2018).

From a logical standpoint, one would expect high citation counts to translate into widespread adoption of MIQE-compliant normalization methods. Yet, a large number of publications still relies on a single-gene approach without a proper validation, with great popularity of “traditional” housekeeping genes (e.g., *ACTB* and *GAPDH*, *RNA18S*), widespread during the days of northern blotting for their ubiquitous and strong expression, yet far from being a “gold standard” (Tricarico et al., 2002; Thellin et al., 1999; Li and Shen, 2013). Congruently, the most recent effort at a systematic review of RT-qPCR techniques concludes that normalization procedures are “inadequate and therefore likely to generate questionable results” (Bustin et al., 2013).

Our direct experience in the field of animal and livestock science is certainly in accordance with this conclusion and cannot but lead to the need for data-driven quantification of this phenomenon. It could be argued that our experience is relevant given two main assumptions: that, as it is often the case, livestock animals often provide a significant molecular model for future larger scale studies in other species (Polejaeva et al., 2016); and that, as the numbers suggest, publications employing RT-qPCR in this field have observed a significant growth in the past decade, with an average annual increase >30% in livestock journals alone in the last 10 years (keywords “Dairy” or “Livestock” or “Poultry” or “Animal” [excluding pets and lab animals] and “RTPCR” or “RT-qPCR” or “RTqPCR”, PubMed search results, 30th August 2018). Therefore, ensuring consistent and reliable quality of RT-qPCR data in livestock science is paramount.

In the earnest effort of raising awareness on this matter, we decided to perform an in-depth meta-analysis of every study that used RT-qPCR in the six most prominent journals in the livestock science over the course of the last five years. Other than a bare assessment of normalization practices in the discipline, we aimed at determining whether the dissemination of reports and criticism over popular normalization methods has had an impact on what is customary in the field, whether this can be measured over time, and how it all associates with other MIQE essential information. We hypothesize that most of the RT-qPCR work in livestock science use unreliable normalization strategies. Our work aims to provide valuable input towards a prospective solution and to reveal potential ground for improvement.

## 2 Materials and methods

The six most relevant journals in livestock science based on their Clarivate Analytics' InCites Journal Impact Factor (**IF**) values for the category “Agriculture, Dairy & Animal Science” were selected for analysis (IF range 1.863–2.474). Keywords “qPCR” and “gene expression” were entered in the advanced search engine of each journal, and only germane primary literature was retrieved. A total of 225 articles published from January 2013 to December 2017 were included after manual verification of fitness, and initially classified based on year, title, and journal of precedence. A detailed analysis of each article was subsequently performed, collecting parameters specified in the MIQE guidelines, including presence or absence of 260/280 absorbance ratio, RNA integrity assessment (presented as RIN or equivalent) and experimental testing of ICG. Relevant observations were collected and presented as a binary qualifier (Yes/No). For each publication, name and number of validated ICG and name and number of ICG selected for normalization were recorded and converted to the HUGO standard nomenclature. A weighted semi-quantitative scale (**SCORE**) was developed based on the following parameters: presence or absence of experimental ICG testing using a validated algorithm (**VALID**; No = 0; Yes = 50); number of ICG selected for normalization (**GENE**; ≤1 ICG = 0; 2 ICG = 23; 3+ ICG = 35); reporting of additional quantifiable output from the normalization software, such as stability values or pairwise variation (**REPORT**; No = 0; Yes = 15). The final value associated with each publication was constructed so that

SCORE=GENE+VALID+REPORT

Consequently, the SCORE ranges from 0 (total noncompliance) to 100 (total compliance). The weight assigned to each fraction is meant to represent the MIQE guidelines' consensus, with experimental validation being the most decisive factor. We recorded other variables from each publication including university/funding agency, species, normalization software/algorithm used, individual PCR efficiency and presence of a standard curve. The complete dataset is available in Supplementary File 1.

The dataset was further divided in two subgroups, based on whether software or an algorithm was employed (**YAL**, *n* = 67) or not (**NAL**, *n* = 158); for both groups, the normalized impact of each ICG was calculated as

$$\frac{\mathrm{CountX}}{\mathrm{CountTOT}}$$

where *Count*_*X*_ represents the count of instances where a gene X was used, and *CountTOT* is the number of unique internal control genes in the subset. A publication-based factor was also generated, dividing *Count*_*X*_ by the number of publications in the subset. For all analyses where publications where not divided in subgroups, technical articles about RT-qPCR normalization (i.e. where the aim of the publications is to determine suitable ICG for some experimental conditions) were omitted to avoid circular reasoning bias.

A simple linear regression was used to determine the effect of Year and Journal on SCORE; models containing the Journal and the Year in different polynomial forms from linear up to effectively treating each year as its own factor were considered. An interaction term between Journal and the functional form of Year was also considered. Akaike Information Criterion (AIC) was used to choose the best model. The model with the lowest AIC had Year as a linear term and no interaction term. The presence or absence of validation, expressed through the VALID component of the calculated score, was considered a binary response, and analyzed through a logistic regression (log link), with Journal and Year as predictors. Similar to SCORE, models containing the Journal and the Year in different polynomial forms from linear up to effectively treating each year as its own factor were considered, along with a possible interaction term, and the logistic model with the lowest AIC was found to have Year as a linear term and no interaction term.

To test whether articles that validated ICG used a different number of genes to normalize than those that did not validate the ICG, a Conway–Maxwell–Poisson distribution was fitted, as a simple Poisson model was significantly underdispersed. As for testing whether genes were used distinctly by articles in the YAL and NAL subcategories, we used Fisher's Exact Test for count data. All statistical analyses and plots were performed in R (version 3.5.1).

## 3 Results and discussion

### 3.1 Poor validation of ICG in livestock-related journals

**Fig. 1:**
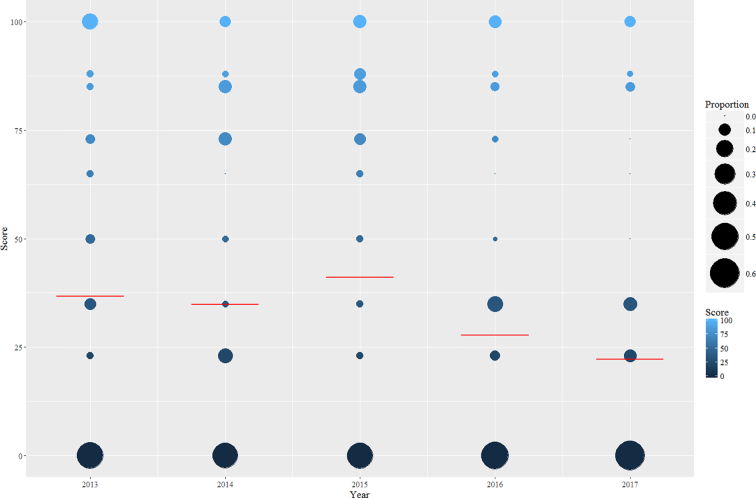
Distribution of SCORE for the surveyed publications over the 2013–2017 period. Bubble size represents the proportion of papers within the subcategory year. Red horizontal lines mark the population mean for each year. (For interpretation of the references to color in this figure legend, the reader is referred to the web version of this article.)

Relative proportions of SCORE in the surveyed period are presented in Fig. 1. Population means range from a minimum of 22.3 in 2017, to a maximum of 41.1 in 2015; furthermore, very few publications obtained a SCORE of 100 (24/225, 10.7%), corresponding to the ideal normalization approach. For practical purposes, SCORE ≥85 could also be considered adequate, as it corresponds to algorithm-driven validation, selecting 3 or more ICG to normalize expression data; however, the cumulative proportion of articles scoring at least 85 is remarkably low (49/225, 21.8%). On the opposite side of the plot, most publications obtained a SCORE = 0, ranging from a minimum of 45.7% of the RT-qPCR manuscripts in 2014 to a maximum of 60.4% in 2017. While numerical variations in population means can be observed in the plot, our model did not identify Year as a significant predictor of SCORE (*p* = 0.69). Journal was identified as a significant predictor (*p* = 0.008); yet, when our model was compared to a slightly more complex one, including an interaction term between the two predictors, a χ^2^ test suggested that while significant differences can be detected between journals in the overall approach, their trend over time is essentially equal (*p* = 0.48). Three relevant inferences can be drawn from these results: first, MIQE-compliant normalization represents a minute proportion of peer-reviewed RT-qPCR publication in the livestock science; second, no tangible improvements seem to have stemmed from numerous reports on widespread subpar normalization and/or providing examples of the proper approach to select ICG (Bustin et al., 2013; Gutierrez et al., 2008a; Guénin et al., 2009; Gutierrez et al., 2008b; Dheda et al., 2004); third, this phenomenon is not characteristic of any journal in particular, as it is replicated similarly by all six journals in our analysis.

**Fig. 2:**
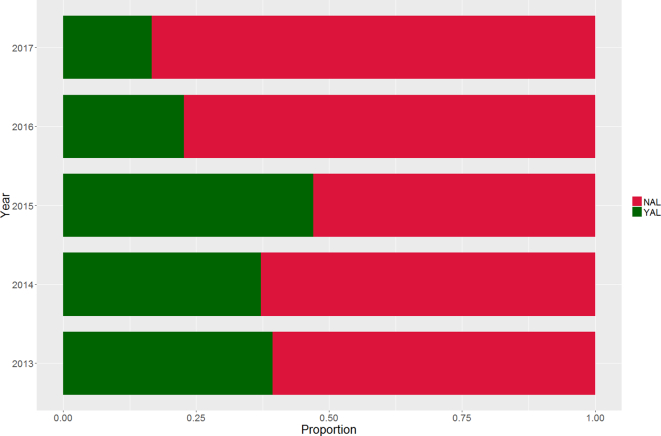
Percentage of publications that selected ICGs using an experimentally validated algorithm (YAL) over the surveyed period.

A similar tendency can be observed in the number of publications validating ICG from a test pool (i.e. using experimentally validated algorithms), expressed as a function of the publications scoring 50 in the VALID portion of SCORE. The percentage of manuscripts validating the ICG using algorithms (presented as YAL) as opposed to the use of citations or unverified classical “housekeeping” genes (NAL) over the surveyed period is reported in Fig. 2. As is immediately evident, proper validation of ICG constitutes a remarkably low proportion, ranging from 16.7% in 2017 to 44.1% in 2015. In this context, a numerical decrease (*p* = 0.11) of data-driven validation was detected in the biennium 2015–2017, where experimental ICG validation decreased from 44.1% in the previous year to 16.7% in 2017.

### 3.2 Few RT-qPCR articles in livestock science use more than one ICG

**Fig. 3:**
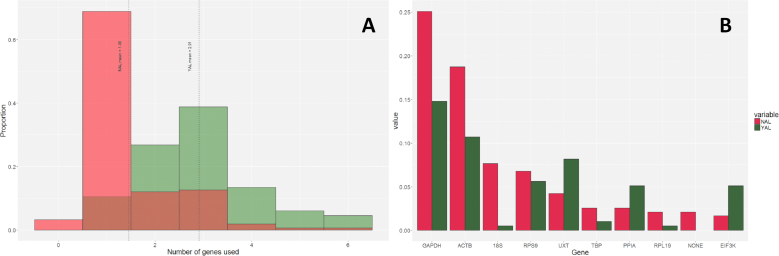
A) Distribution of ICG used for normalization in the YAL and NAL subgroups. B) Frequency of appearance of the eight most used ICG to normalize RT-qPCR data for the YAL and NAL subgroups, relative to the total number of genes tested in each subgroup. Genes of internal control gene tested are reported. NONE = no internal control genes were tested.

Dividing articles in the YAL and NAL subgroups, allowed assessing the effect of utilizing software on the number and type of ICG that were selected for normalization. The use of experimentally-validated algorithms to select ICG was significantly associated (*p* = 1.37 × 10^−13^) with a larger number of ICG used to calculate the normalization factor (Fig. 3A): publications in the subgroup YAL utilized 1.44 more genes (95% C.I.: 1.11–1.84) than those in NAL. The immediate implication is that using one gene, as 75.6% of the publications in NAL did, does not guarantee compliance with the MIQE guidelines. The guidelines were reasonably designed around several reports reasoning on how the trend of multiple ICG is significantly more likely to provide a reliable normalizer than one ICG alone. As an example, Vandesompele et al. manifest how the stability value M, displayed as part of the output of geNorm, declines with the inclusion of more genes in the normalizing factor, which in turn provides a more robust means to account for extrinsic variation (Vandesompele et al., 2002). Another important consideration is tied to these results: despite multiple publications still justifying the use of a single ICG whose expression is not significantly altered through time or sample subgroup, it has been demonstrated that in several experimental conditions the invariance of a gene must be considered a stochastic artifact. This is notably the case of tissues whose transcriptome is undergoing drastic changes (e.g. mammary tissue or liver during the transition between pregnancy and parturition): for a set amount of purified RNA, a large upregulation of key transcripts will “dilute” the bulk of measurable cDNA, causing stable genes to appear as downregulated (Reinhardt and Horst, 1999; Bionaz and Loor, 2007; Xu et al., 2015). Algorithms using a pairwise approach to validate ICG described herein, and used by packages such as geNorm and BestKeeper, can account for said effect by detecting similar trends of gene expression ratio that can be affected by the artificial dilution effect in otherwise stable transcripts. It will thus appear patent that declaring an ICG stable based on statistical stability by following its isolated trend does not guarantee true invariance. In this context, our results underline that authors who utilized data-driven validation of their RT-qPCR assays are significantly more likely to avoid biases due to artifacts.

### 3.3 The use of algorithms to assess RT-qPCR normalization diversifies ICG choice and reveals that frequently used housekeeping genes are seldom suitable

An additional case must be made for the choice of unique ICG, as the routine usage of *GAPDH*, *ACTB*, *RNA18S*, the three most common ICG, has been demonstrated to provide unreliable results when used individually (Selvey et al., 2001; de Jonge et al., 2007; Lisowski et al., 2008; Ruan and Lai, 2007). As expected, these three “classical” ICG were by far the most frequently employed, considering that 76.4% of all the publications in the dataset used at the least one of the three. Both YAL and NAL made similar use of *GAPDH* (43.3% vs. 37.3%, *p* = 0.45) and *ACTB* (31.3% vs. 27.8%, *p* = 0.87) on a per-publication basis, whereas publications in YAL used *RNA18S* significantly less (1.5% vs. 11.4%, *p* = 0.02) than their NAL counterpart. Conversely, when a weighted approach is implemented by scaling individual instances of an ICG to the total number of genes in the subset rather than the number of articles, a considerably lower impact of the aforesaid ICG in the YAL group than in NAL is detected (Fig. 3B). This can partly be attributed to the fact that more unique ICG where used in the YAL dataset than in the NAL dataset (64 vs. 50, respectively), despite the latter having more publications (72 vs. 156, respectively).

From a hypothetical standpoint, almost every gene can be considered a “candidate” ICG. As we claimed that no ICG should be appointed without validation, the reverse is also true: no gene can be deemed unsuitable without undergoing an adequate validation process. Earlier in this manuscript we discussed how three “housekeeping” genes are often employed indiscriminately as ICG across the board, as well as by a vast number of publications in our analysis. Albeit often considered stable, housekeeping genes have been reported to adopt erratic expression patterns in certain experimental conditions, or to be directly or indirectly regulated by the treatments. Relevant to the aforementioned examples, *ACTB* is variably expressed in human prostate cancer (Ohl et al., 2005), mouse brain (Bonefeld et al., 2008) and *A. flavicollis* (Axtner and Sommer, 2009), while *GAPDH* patterns are too unstable in adipose stem cells (Fink et al., 2008), PC12 cells (Zhou et al., 2010), and samples derived from patients suffering from tuberculosis (Dheda et al., 2005), among others. Even more criticism has been attributed to the use of *RNA18S*, where fluctuations in the rRNA:mRNA ratios have been reported (Solanas et al., 2001), as well as effects on ribosomal RNA transcription in response to drugs and experimental treatments (Spanakis, 1993; Nicot et al., 2005). Further, two crucial drawbacks are associated with the use of ribosomal RNA as a normalizer: on the one hand, 18S RNA lacks a poly(A) tail, and is thus commonly transcribed independently from poly(dT)-primed transcripts, which generates further bias (Brunner et al., 2004); on the other hand, ribosomal cDNA is generally several orders of magnitude more abundant than its mRNA counterpart, which requires that it be diluted, introducing an additional source of technical variability (Wong and Medrano, 2005).

The use of an algorithm, as specified earlier, conveniently generates a stability value for the examined subset of candidate ICG. This, in concurrence with extensive reporting of both the ICG that were tested and those that were deemed suitable by the algorithm, allowed us to assess the overall reliability of the most common ICG in the subset. YAL publications in which *GAPDH*, *ACTB* or *RNA18S* underwent experimental validation were analyzed, as well as the outcome of said validation for each. Both *GAPDH* and *ACTB* were not considered adequate ICG in around 25% of the surveyed publications, whereas *RNA18S* was rejected by 75% of the qualifying articles (Table 1). While extending these results to the publications in NAL would be mere speculations, it undeniably adds to the gravity of our findings.

**Table 1:** Outcome of validation procedures of *GAPDH*, *ACTB* and *RNA18S* in the YAL subset when considering the number of articles where these were tested and selected.

Gene	Tested	Selected	Ratio (Selected/Tested)
GAPDH	38	28	73.7%
ACTB	29	22	75.9%
RNA18S	8	2	25.0%

### 3.4 The use of algorithms to assess RT-qPCR normalization improves MIQE compliance

As an additional measure of good quality, we wanted to determine how the use of an algorithm for normalization relates with other parameters regarded as “Essential Information” by MIQE. To guarantee compliance with the guidelines, publications are required to report the absence of protein or phenol contaminants in their RNA samples (through the 260/230 and 260/280 ratios) and RNA integrity, either verified via gel electrophoresis or using microfluidics instruments and reported as RIN, RQI, RIN^e^ or equivalent. RNA purity and integrity are particularly relevant in our approach, as they can constitute a hallmark of high exogenous variation by means of hindering reaction efficiency (low purity (Ning et al., 2009)) or reducing amplification affinity/specificity (low integrity (Fleige et al., 2006)) in a specific experimental unit. When comparing the two subgroups in our analysis, both performed similarly in reporting purity (1.5% vs. 2.5%, *p* = 0.63); however, a significantly higher percentage of publications in the YAL subgroups reported RNA integrity values (70.1% vs. 32.1%, *p* = 3.16 × 10^−7^) than those in NAL. An immediate explanation of these findings is challenging, as there is no procedural link between normalization and integrity assessment (i.e. they are separate tasks). Nevertheless, we could suggest that greater care in the choice of normalization strategies stems from greater observance of the guidelines altogether, and said care is likely to be applied to other unrelated parameters (as is the case with RNA integrity).

### 3.5 From data to practice: resource-efficient ICG validation

Selecting and validating candidate ICG can be a recursive and expensive process, as often the researcher will have little to no information to construct the initial pool of candidate ICG. Despite its undeniable importance introduced earlier, it is thus key that those engrossed in any RT-qPCR endeavor maximize their time and effort in achieving reliable results. Preceding larger-scale transcriptomic studies can significantly reduce the effort required in determining size and components of the candidate gene test pool; multiple studies indeed utilize RT-qPCR when further sensitivity is required after microarray or RNAseq studies, and thus locate the most stable genes among those results (Kadegowda et al., 2009b; Popovici et al., 2009). When said results are not accessible, or not required by the scope of the study, the literature can provide a valuable tool in locating transcriptomic studies similar in goals or biological samples. A quick summary of the most validated genes in bovine studies in our analysis is included with this manuscript (Supplementary Table 1), and can offer a starting point for the construction of a test pool of reference genes. Increasingly systematic approaches will reduce the tediousness of this trial-and-error process; such is the work of Sang and collaborators, who have developed an open-sourced and publicly editable database of validated ICG in several organisms (ICG, available at http://icg.big.ac.cn/index.php/Main_Page) (Sang et al., 2018). The knowledgebase has rapidly grown in size since its introduction, and it is bound to continue to do so. Researchers can consult it for a list of candidate ICG for each species and in particular experimental conditions, complete with information on how they were measured and primer sequences. The database also contains general knowledge on the most popular normalization algorithms and how to use them.

Regarding algorithms, we wish to stress the importance of cross-referencing outputs of several of them. If co-regulated ICG are properly excluded from validation, all algorithms should generate comparable outputs: reports have shown similar or identical suggestions between geNorm pairwise approach and NormFinder output even in large and heterogeneous databases (Willems et al., 2004). In any case, public normalization software packages provide a ranking of candidate ICG with a stability value associated with each: it is thus key that the author's instructions for each algorithm are used as a reference to adequately select ICG. Ultimately, the author of this manuscript cannot vouch for any one approach, as despite the differences between them a significant body of literature exists comparing benefits and drawbacks of each. It is however fundamental that option of choice be declared, and detailed results of the validation process be reported, including number and name of candidate ICG, their respective stability value and supplementary information as needed. Contribution to open-source projects can be an effortless way to keep a manuscript's narrative streamlined while still significantly contributing to solve this issue.

## 4 Conclusions

In line with previous reports, our results underscore that normalization of RT-qPCR research in livestock science is well below acceptable thresholds. Further, our analysis confirms that the use of widely-cited and well-established validation algorithms increases the number and variety of ICG used for normalization, while guaranteeing accurate and data-driven assessment of stability. This, widely regarded as the most effective method for normalization, is key in ensuring that exogenous sources of variation are not overlooked. Although several efforts at addressing the issue of poor normalization have been published over the last decade, it is arguable that the key to community awareness lies in the editorial process. Editors and reviewers alike should be familiar with the MIQE guidelines, and extensive and detailed reporting of the normalization strategies should be a strict requirement for publication of every RT-qPCR endeavor. It is our community's utmost responsibility to minimize confounding factors and provide the community with the truest representation of nature that the method will allow; willfully ignoring sources of bias is certainly not in harmony with our moral duties.

The following are the supplementary data related to this article.

Supplementary File 1 Complete dataset of publications included in the manuscript. Supplementary File 1

Supplementary Table 1 Ten most validated genes in bovine RT-qPCR studies, ranked by their usage over the total of genes. Supplementary Table 1

## Conflict of interest statement

The authors declare no conflict of interest.
