# Supplementary material for: What's the norm in normalization? A frightening note on the use of RT-qPCR in the livestock science
Source: Gene X. 2018 Dec 23;1:100003. doi: 10.1016/j.gene.2018.100003 (PMC7285961; doi:10.1016/j.gene.2018.100003)
Supplement: Supplementary Table 1 — Ten most validated genes in bovine RT-qPCR studies, ranked by their usage over the total of genes. [file mmc2.docx]

| Gene | Instances | Ratio |
| --- | --- | --- |
| GAPDH | 19 | 15.83% |
| ACTB | 11 | 9.17% |
| RPS9 | 10 | 8.33% |
| UXT | 7 | 5.83% |
| EIF3K | 5 | 4.17% |
| SDHA | 5 | 4.17% |
| RPS15A | 4 | 3.33% |
| PPIA | 3 | 2.50% |
| ATP5B | 3 | 2.50% |
| OSBPL | 2 | 1.67% |
| **Total** | 120 | 100 % |

**Supplementary Table 1**. Ten most validated genes in bovine RT-qPCR studies, ranked by their usage over the total of genes.
